# An Effective Clustering Algorithm Using Adaptive Neighborhood and Border Peeling Method

**DOI:** 10.1155/2021/6785580

**Published:** 2021-11-10

**Authors:** Ji Feng, Bokai Zhang, Ruisheng Ran, Wanli Zhang, Degang Yang

**Affiliations:** ^1^College of Computer and Information Science, Chongqing Normal University, Chongqing 401331, China; ^2^Chongqing Engineering Research Center of Educational Big Data Intelligent Perception and Application, Chongqing Normal University, Chongqing 401331, China

## Abstract

Traditional clustering methods often cannot avoid the problem of selecting neighborhood parameters and the number of clusters, and the optimal selection of these parameters varies among different shapes of data, which requires prior knowledge. To address the above parameter selection problem, we propose an effective clustering algorithm based on adaptive neighborhood, which can obtain satisfactory clustering results without setting the neighborhood parameters and the number of clusters. The core idea of the algorithm is to first iterate adaptively to a logarithmic stable state and obtain neighborhood information according to the distribution characteristics of the dataset, and then mark and peel the boundary points according to this neighborhood information, and finally cluster the data clusters with the core points as the centers. We have conducted extensive comparative experiments on datasets of different sizes and different distributions and achieved satisfactory experimental results.

## 1. Introduction

In real-world, high-dimensional data lie close to low-dimensional structures corresponding to several classes or categories to which the data belong, and clustering is an efficient way to solve this problem. The goal of clustering is to unsupervisedly partition datasets into different classes or clusters so that the similarity of data objects within the same cluster is as large as possible, while ensuring that the differences between data objects in the clusters are also as large as possible. Among many clustering algorithms, the nearest neighbor-based clustering algorithm has been widely used in many fields, such as data mining, machine learning, image processing, and pattern recognition, and has achieved many good results.

In nearest-neighbor-based clustering algorithms, it is a challenge for clustering algorithms to automatically infer the number of clusters and neighborhood parameters to reduce the dependence on a priori knowledge. In order to obtain more accurate clustering results, existing clustering algorithms generally need to specify the number of clusters in advance. In real-world applications of clustering methods, it is difficult to estimate the number of clusters accurately before the clustering algorithm is performed. On the other hand, either based on the *k*-nearest neighbor (KNN) principle or the *ε*-nearest neighbor (*ε*-NN) principle, the neighborhood parameters *k* and *ε* are closely related to the distribution characteristics of the dataset, and the performance of the algorithm can change drastically depending on the set of different values.

**Figure 1:**
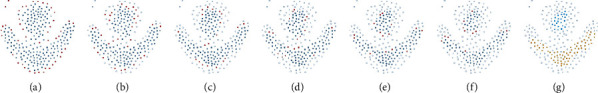
(a)–(f) The clustering process of SANBP algorithm. (g) The clustering result of the separable core points. Border points are identified by red colour.

For the above parameter selection problem, we propose an effective clustering algorithm (self-adaptive neighborhood border peeling clustering algorithm (SANBP)). SANBP algorithm is based on natural neighbor method and proposes logarithmic natural neighbors that are more applicable to the border peeling method. Therefore, the algorithm solves the neighborhood parameters selecting problem, while adaptively obtaining different neighborhood sizes for each data point. In the process of border peeling, the algorithm can get more accurate initial boundary points according to the logarithmic natural stable state and also can obtain more accurate distribution information during the peeling process by the adaptive neighborhood. Therefore, the algorithm can obtain clustering results that are more consistent with the actual distribution without the need to set prior parameters artificially.  Figure 1 illustrates the clustering process of our algorithm.

The main contributions of this paper are as follows:

1. We propose a more stable concept of logarithmic stable state based on the natural neighborhood.
2. A self-adaptive neighborhood border peeling clustering algorithm is proposed. This algorithm is able to perform adaptive clustering on different shaped datasets, substantially improving the adaptive nature of the algorithm.
3. SANBP algorithm is capable of clustering datasets with different densities and distributions and adaptively determines the number of clusters and neighborhood sizes.

## 2. Related Work

### 2.1. Natural Neighbors

The nearest neighbor idea is widely used in clustering algorithms, and almost all clustering algorithms use the nearest neighbor idea in one way or another, and their core methods are based on KNN and *ε*-NN [1]. Both methods use neighborhood parameters, which can only be determined empirically or by multiple attempts, and are heavily dependent on the data distribution. To address this problem, the natural neighbor approach proposes a new idea to solve the parameter problem based on the combination of nearest neighbor and reverse nearest neighbor.

Natural neighbor (NaN) is a new concept of neighbor, which arises from the perception of objective reality [2, 3]. Natural Neighbor is different from KNN and *ε*-NNN the biggest difference is natural neighbors do not need to set or fix a certain parameter *k* or. As an alternative to *k*, natural neighbors are calculated adaptively, and the number of natural neighbors of each data object in the dataset is not the same, so the natural neighbor is a scale-free neighbor concept [4].

Nowadays, the idea of incorporating the concept of natural neighbors into clustering algorithms has had many achievements and has good experimental results in various fields, such as hierarchical clustering algorithms based on noise removal [5], adaptive spectral clustering algorithms based on natural neighborhoods [6], and clustering methods based on natural neighbors [7, 8]. Two types of nearest neighbor KNN [9]and reverse *k*-nearest neighbor (RKNN) [10]search algorithms are also widely used in the above clustering algorithms.

### 2.2. Clustering Algorithm

As the focus on data analysis has gradually increased, more and more clustering algorithms have been proposed. Among them, the core idea of the division-based clustering algorithm is to divide the dataset into several classes according to the global optimization criteria. However, due to the limitation of the global optimization function of the division-based clustering algorithm itself, it is not applicable to many problems such as stream-shaped and concave datasets. The density-based clustering algorithm is theoretically able to apply to any shaped dataset, but it is more sensitive to parameters and is not applicable to datasets with high density or complex stream shapes [11]. The core idea of hierarchical-based clustering algorithm is to calculate the similarity between nodes by some similarity measures and to gradually reconnect the nodes by sorting them from highest to lowest similarity [12, 13]. The advantage of hierarchical clustering is that the similarity of distances and rules is easy to define and less restrictive and does not require a predetermined number of clusters, but the complexity of hierarchical clustering is high and singular values can have a large impact. The spectral clustering algorithm contains rigorous mathematical logic to partition the dataset by graph partitioning and is theoretically able to solve the streamlined data problem [14] and can even handle extremely large-scale datasets with limited resources [15]. Sparse subspace clustering, on the other hand, is another important branch of research in clustering problems, and a series of successful studies on this problem have been carried out by Elhamifar and Vidal [16]. Lu et al. extend the spectral clustering with sparse regularization [17], and He et al. improved L_1_-minimization by a new projection neural network to solve the sparse signal reconstruction problem in sparse subspace clustering [18].

Among the above clustering algorithms, the density-based clustering algorithm is closer to the daily application scenarios in terms of clustering results, and researchers have proposed a large number of improved algorithms for different application areas. Rodriguez proposed a novel CFDP clustering algorithm in density clustering algorithm, which can describe the density peak clustering more accurately and quickly [19]. Later, based on DBSCAN [20], Ding proposed a new density-based OPTICS clustering algorithm [21]. The OPTICS clustering algorithm mainly improves the density-based clustering algorithm on the problem of parameter sensitivity and reduces the sensitivity of the algorithm to parameters. The grid-based algorithm mainly scans the dataset by dividing the data space into several grid cells according to the selected attributes and dividing the sample points into the corresponding cells and finally forming class clusters based on the density of the cells [22]. Since the final clusters are divided according to the grid cells, they are very sensitive to the density threshold, and it is easy to lose the class clusters. When there are clusters with different densities in the dataset, a high threshold setting may lose part of the clusters, and a low one may make the two class clusters that should be separated merge. To improve the effectiveness of density-based clustering algorithms, Huang completed the QCC clustering algorithm by finding the centroids based on the cluster centers method, using RKNN clustering, and the results are obtained from the cluster centers determined by the algorithm [23]. Campello proposed the HDBSCAN clustering based on the previous DBSCAN and OPTICS algorithm; the algorithm requires only a minimum cluster parameter and is able to automatically select a density threshold but is not sensitive enough to noisy points [24]. Cheng et al. proposed the mean-shift method for clustering data points using the kernel density estimation function, which iteratively moves each data point to its neighbor dense region and then clusters the moved data points, but their algorithm often relies on the bandwidth parameter of the kernel density estimator [25]. Shimshoni et al. proposed the adaptive mean-shift method to overcome this problem by estimating a different bandwidth for each data point based on the local neighborhood of each data point, but this method is usually prone to over-clustering of the data [26]. Averbuch-Elor et al. used the border peeling idea to propose a novel centroid-based border peeling clustering algorithm and achieved excellent clustering results [27].

However, the initial boundary points selected by the border peeling clustering algorithm on different shaped datasets are extremely dependent on the selection of the neighborhood parameter *k*, which makes the process of boundary points from boundary points to core points in the process of iterative peeling have the possibility of producing deviations, which affects the clustering results and even shows extremely unreasonable data cluster partitioning in some datasets. Based on the above problems, this paper proposes a new algorithm combining natural neighbors and border peeling clustering algorithm, SANBP, which can retain the advantages of the original border peeling clustering but also make up for the shortcomings of the border peeling clustering algorithm that always has the neighborhood parameter and can be used on datasets of different shapes without setting the neighborhood parameter. It can obtain clustering results that match the characteristics of data distribution adaptively without setting the neighborhood parameters on different shaped datasets.

## 3. Materials and Methods

The natural neighbors used in this paper are a scale-free concept, and natural neighbors do not require parameters in the selection of neighbors.

### 3.1. Basic Natural Neighbor Method

The following is a precise description of natural neighborhood method through the definition of the relevant concepts.

Dataset *X*={*x*_1_, *x*_2_, *x*_3_,…, *x*_*n*_} where *n* is the total number of points in the dataset *X*.

**Definition 1 (natural neighborhood).**

When the dataset is in a natural stable state, the points that are neighbors of each other are natural neighbors of each other.

(1) $$\begin{array}{c} x_{j}\in \text{NaN}\left(x_{i}\right)\Leftrightarrow \left(x_{i}\in {\text{KNN}}_{\lambda }\left(x_{j}\right)\right)\wedge \left(x_{j}\in {\text{KNN}}_{\lambda }\left(x_{i}\right)\right). \end{array}$$

**Definition 2 (natural stable state).**

The natural neighbor searching process reaches natural stable state only if all points have at least one mutual neighbor, when the searching round *r* increases from 1 to *λ*.

**Definition 3 (natural eigenvalue).**

When the dataset *X* is in the natural stable state, the natural neighborhood eigenvalue is the current searching round *λ*. The natural eigenvalue is the maximum number of cycles in the actual running process, and *λ* reflects the distribution pattern of the dataset.

### 3.2. Basic Principles of Border Peeling Clustering

By given the dataset *X* and the artificially set neighborhood parameter *k*, the neighborhood parameters of KNN and RKNN are first adopted by the natural eigenvalues; for any point *x*_*i*_*εX*^(*t*)^, KNN_*r*_^(*t*)^(*x*_*i*_) denotes the set of *k*-nearest neighbors; the RKNN of *x*_*i*_ is defined as

(2) $$\begin{array}{c} {\text{RNN}}_{r}^{\left(t\right)}\left(x_{i}\right)=\left\{x_{j}|x_{i}\varepsilon {\text{KNN}}_{r}^{\left(t\right)}\left(x_{j}\right)\right\}. \end{array}$$

Each point will be associated with a density impact value; through the density impact value, this paper hopes that the closer to the center of the cluster, the greater the value, for points far from the center of the cluster, the smaller the value. The core of border peeling clustering lies in continuously iterating to determine the boundary points and continuously peeling them until finally establishing the core points. For each *x*_*i*_ ∈ *X*^(*t*)^, using *B*_*i*_^(*t*)^ to denote the value of boundary of each point,

(3) $$\begin{array}{c} B_{i}^{\left(t\right)}\left\{\begin{array}{cc} 1, & \text{if }b_{i}^{\left(t\right)}\leq \tau^{\left(t\right)},\\ 0, & \text{otherwise.} \end{array}\right) \end{array}$$

To control the end of iterative peeling, the set of boundary points is defined as

(4) $$\begin{array}{c} X_{B}^{\left(t\right)}=\left\{x_{i}:B_{i}^{\left(t\right)}=1\right\}. \end{array}$$

The next set of unpeeled boundary points is defined as

(5) $$\begin{array}{c} X^{\left(t+1\right)}=\frac{X^{\left(t\right)}}{X_{B}^{\left(t\right)}}. \end{array}$$

After identifying each boundary point, each boundary point is associated with a nearest nonboundary point, using the association node *ρ*_*i*_ ∈ *X*^(*t*+1)^, and then, the border peeling process marks some points as outliers, which do not belong to any cluster.

(6) $$\begin{array}{c} \rho_{i}=\left\{\begin{array}{cc} x_{j}, & \delta \left(x_{i},x_{j}\right)\leq l_{i},\\ \emptyset , & \delta \left(x_{i},x_{j}\right)>l_{i}, \end{array}\right) \end{array}$$

where *l*_*i*_ is a variable threshold, *xi* is a boundary point, and *xj* is the closest point to *xi* in the set of nonboundary points. If the distance between xi and *xj* is greater than *li*, *xi* will be marked as an outlier. And within the variable threshold, *ρ*_*i*_ is the nearest nonboundary point of *x*_*i*_.

Finally, after several iterations of peeling the boundary points, the final remaining nonboundary points are the core points, each core point has a transfer association to the initial boundary point. By the method of literature [26], each pair of reachable core points is gradually merged, and finally the candidate class clusters are defined by associating and linking the boundary points with the core points. Also, to better identify outlier points, BP algorithm marks small clusters as noise.

### 3.3. Self-Adaptive Neighborhood Border Peeling Clustering Algorithm (SANBP)

For a given dataset *X*, the algorithm will first perform a natural neighbor search based on logarithmic stable state. After the natural neighbor search step, each data point in the dataset will be given an adaptive neighborhood. The neighborhood of each point is adaptively calculated by the algorithm based on the density of its distribution, which varies in size and can provide more accurate density information in the next step. Secondly, the algorithm will obtain the initial boundary points based on the logarithmic natural eigenvalue and then gradually complete the boundary point peeling based on the different neighborhood sizes of the points. Finally, the algorithm will cluster the core points as the center to obtain the clustering results of the dataset without the number of clusters.

**Definition 4 (noise point-NOS).**

In the process of natural neighbor search in the dataset, when the number of search iterations reaches *λ*, the point which is no nearest neighbor for any data point is noise point, which is defined as

(7) $$\begin{array}{c} x_{i}\in \text{NOS}\Leftrightarrow {\text{KNN}}_{\lambda }\left(x_{i}\right)=\emptyset . \end{array}$$

**Definition 5 (logarithmic natural stable state).**

Given a dataset *X* = {*x*_1_, *x*_2_, *x*_3_,…, *x*_*n*_}, during the natural neighborhood search process, if the number of noise points in the dataset remains constant over multiple consecutive rounds, and its value is greater than the natural logarithm of the dataset, then the current neighborhood state of the data is the logarithmic natural stable state, which is defined formally as follows:

(8) $$\begin{array}{c} \forall \left(x_{i},x_{j}\right)\in \text{NOS}\Rightarrow \left(x_{j}\notin {\text{KNN}}_{\lambda }\left(x_{i}\right)\right)\wedge \left(x_{j}\notin {\text{KNN}}_{\lambda +\ln\;n}\left(x_{i}\right)\right). \end{array}$$

**Definition 6 (logarithmic natural eigenvalue).**

When the dataset *X* is in the logarithmic natural stable state, for the logarithmic natural stable state, we propose the logarithmic natural eigenvalue, which is defined as follows:

(9) $$\begin{array}{c} r={\left(\lambda +\ln\;n\right)}_{\lambda \in N,\;\ln\;n\in N}\left\{\lambda |\forall \left(x_{i},x_{j}\notin \text{NOS}\right)\wedge \exists \left(x_{j}\in {\text{KNN}}_{\lambda +\ln\;n}\left(x_{i}\right)\right)\wedge \left(x_{i}\neq x_{j}\right)⟶\exists \left(x_{j}\in {\text{KNN}}_{\lambda +\ln\;n}\left(x_{i}\right)\left(\right)\right)\right\}, \end{array}$$

where *λ + *ln *n* denotes the round of the natural neighbor search algorithm process and logarithmic natural eigenvalue according to the distribution characteristics of the dataset and also can be used as a reference for the traditional KNN neighborhood parameters.

**Definition 7 (logarithmic natural neighbors).**

When the dataset is in a logarithmic natural stable state, points that are neighbors of each other are logarithmic natural neighbors of each other.

(10) $$\begin{array}{c} x_{j}\in \text{NaN}\left(x_{i}\right)\Leftrightarrow \left(x_{j}\in {\text{KNN}}_{\lambda }\left(x_{i}\right)\right)\wedge \left(x_{i}\in {\text{KNN}}_{\lambda }\left(x_{j}\right)\left(\right)\right). \end{array}$$

The number of log natural neighbors for each point is not necessarily the same, and the number of neighbors depends on the distribution of the dataset, and the algorithm is able to find the appropriate number of neighbors for each point according to the distribution of the dataset.

Here, findKNN (*x*_*i*_, *r*, *T*) returns the *r*-th neighbor of point *x*_*i*_ in *k* − *d* tree *T*, NaN*_*Edge means the edge set of natural neighbor, each edge connects two natural neighbor points, and NaN*_*Num (*x*_*i*_) means the natural neighbor number of point *x*_*i*_. The natural neighbor search algorithm first finds one neighbor for each data point, then calculates the number of points in the dataset whose mutual neighbor point is zero, and then finds two neighbors for each data point to calculate the number of points in the dataset whose mutual neighbor point is zero. The algorithm keeps increasing the number of neighbors for each data point and then calculates *ξ* as the threshold value. If the number of points with zero mutual neighbors does not change in *ξ* round, the algorithm determines that the current search has reached the logarithmic natural stable state.

**Figure 2:**
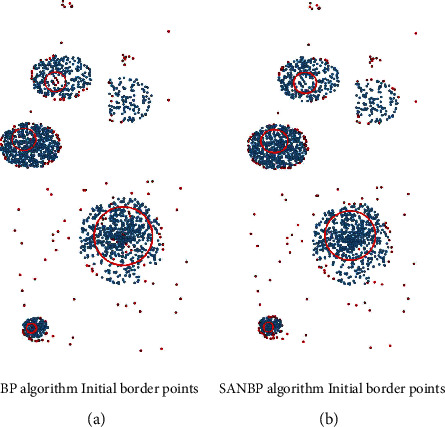
The left column represents the initial border point determined using the fixed neighborhood parameter *k* = 20; the right column represents the initial border point determined using SANBP algorithm without parameter. (a) BP algorithm initial border points. (b) SANBP algorithm initial border points.

Figure 2 demonstrates the superiority of the initial boundary point selection in SANBP algorithm. Within the red circle marked in the figure, it can be intuitively seen that it is in the cluster center position, which should obviously be a candidate for core points and should not be marked as edge points by the current step. The boundary points determined by SANBP algorithm are basically zero in these places, while the original fixed neighborhood parameters appear to have more unreasonable boundary points in this part. Figure 2 graphically demonstrates that the initial boundary points generated using SANBP algorithm are more reasonable than the BP clustering algorithm on datasets of different shapes. The initial boundary points, excluding the noisy points far from the class clusters, are basically reasonably distributed at the class cluster edges. In contrast, the initial boundary points of BP clustering algorithm on different shaped datasets are not well determined, which leads to insufficient self-adaptive ability to the dataset and then seriously affects the selection of core points in the subsequent algorithm.

**Definition 9 .**

(similarity metric). To estimate distances between points, we use Euclidean distance as the dissimilarity measure, and apply a Gaussian kernel *σ*_*j*_ to construct the function *f*:

(11) $$\begin{array}{c} f\left(x_{i},x_{j}\right)=\exp\left(-\frac{x_{i}-x_{j}{\;}_{2}^{2}}{\sigma_{j}^{2}}\right). \end{array}$$

**Definition 10 .**

(density influence value). After determining the similarity metric, we associate each point with a density influence value and use the number of natural neighbors to generate the density impact value *b*_*i*_^(*t*)^. Because different data points have different numbers of natural neighbors, the algorithm can obtain density impact values with more local feature information and measure the relationship between data points and clustering centers by their magnitude.

(12) $$\begin{array}{c} b_{i}^{\left(t\right)}=\underset{x_{j}\in {\text{RNN}}_{\text{NaN}\_\text{Num}\left(x_{i}\right)}^{\left(t\right)}\left(x_{i}\right)}{\sum }f\left(x_{i},x_{j}\right). \end{array}$$

The core steps of the whole algorithm consist of the following two parts: (1) the adaptive dataset reaches logarithmic natural stable state and generates logarithmic natural eigenvalues; (2) the natural neighbor numbers of points are used to establish reasonable initial boundary points and perform border peeling clustering. The steps are described in detail in the pseudocode of Algorithms 1 and 2. SANBP algorithm firstly changes the defects of the inherent neighborhood parameters of the original BP clustering algorithm. The algorithm uses a robust natural search algorithm to bring the dataset to a logarithmic natural stable state, which can generate different neighborhood according to different shapes of the dataset and every point in the datasets. On this basis, the algorithm replaces the original BP clustering algorithm neighborhood parameter *k* = 20 with self-adaptive neighborhood. Secondly, the neighborhood parameters obtained by SANBP algorithm can better adapt to the distribution pattern of the dataset and can establish good initial boundary points in the process of iterative peeling of boundary points. In the process of boundary point peeling, the establishment of initial boundary points has a great influence on the final clustering effect of different shaped datasets. The BP clustering algorithm uses inherent neighborhood parameters, and the adaptive ability of initial boundary point establishment is obviously insufficient when facing different shaped datasets. SANBP algorithm solves this problem well and demonstrates its superiority graphically in the subsequent experiments.

## 4. Results and Discussion

In order to evaluate the natural neighbor-based border peeling clustering algorithm to be more generalizable, six datasets with different shapes (flame, R15 from literature [26], compound, D31, data_DBSCAN, and artificial data from literature [3]) were selected and tested on this paper, and their performance was compared with the border peeling clustering algorithm which was compared to the experiments. The final clustering effect of the boundary peeling clustering algorithm relies heavily on the determination of the initial boundary point, which is directly related to the neighborhood parameter *k*. Therefore, the experiments on the boundary peeling clustering algorithm for different shaped datasets in this paper still retain the original fixed parameters. In contrast, SANBP algorithm proposed in this paper does not use fixed neighborhood parameters, and the variable neighborhood obtained by adaptive data features can make the initial boundary points in border peeling better distributed, and it can show good results on different shaped datasets.

The comparison experiments include four datasets with different shapes and quantities in addition to the dataset used in the BP algorithm. In order to better demonstrate the characteristics of the algorithm in this paper, high-dimensional large data are selected for experimental comparison in Section 4.3.

### 4.1. Experiments with Supervised Datasets

In order to verify the superiority of the algorithm proposed in this paper, the artificial datasets used in the BP clustering algorithm were selected for the experimental step for comparison tests. The four datasets selected for the experiments are with real labels and the evaluation metrics are ARI and AMI.

ARI is a similarity metric describing the randomly assigned cluster class marker vector defined as

(13) $$\begin{array}{c} \text{ARI}=\;\frac{\text{RI}-E\left[\text{RI}\right]}{\max\left(\text{RI}\right)-E\left[\text{RI}\right]}. \end{array}$$

AMI is based on the mutual information score between the predicted cluster vector and the real cluster vector to measure its similarity; the larger AMI indicates higher similarity. AMI is defined as

(14) $$\begin{array}{c} \text{AMI}=\frac{\text{MI}\left(U,V\right)-E\left\{\text{MI}\left(U,V\right)\right\}}{F\left(H\left(U\right),H\left(V\right)\right)-E\left\{\text{MI}\left(U,V\right)\right\}}. \end{array}$$

**Figure 3:**
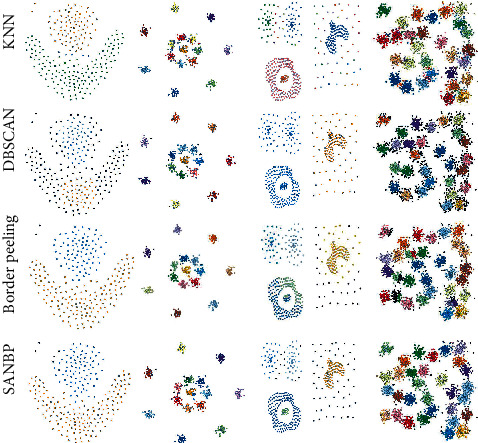
Comparison with BP clustering algorithm on flame, R15, compound, and D31 datasets.

The experimental results on the datasets (flame, R15, compound, and D31) are shown in Figure 3. SANBP-based method used in this paper generates natural neighborhood instead of *k* values through the idea of natural neighbors. The experimental results of compound dataset show that our method can distinguish the class clusters of different shaped datasets well, while the D31 dataset shows that our method is more reasonable in determining the outliers.

Table 1 lists the evaluation metrics for the four datasets. In order to better demonstrate the superiority of our algorithm, we conduct comparison experiments by having the comparison algorithms run multiple rounds with different parameter settings, respectively, and select the best results for comparison with our SANBP algorithms. For the first two datasets (flame and R15), it can be intuitively seen that SANBP clustering method proposed in this paper can still perform well on the two different shaped datasets used in the original paper, and more importantly, SANBP method can adaptively generate different log natural eigenvalues for the different shaped datasets, while showing better results in the ARI and AMI evaluation metrics. For the latter two datasets (compound and D31) in Figure 2, it can be visualized that the log natural eigenvalues generated by SANBP method on the other two supervised datasets of different shapes, compound and D31, both adapt well to the data distribution pattern and exceed the original BP clustering algorithm in both ARI and AMI evaluation metrics, indicating the good adaptive power of the proposed method for the different shaped datasets in this paper. DBSCAN algorithm is more sensitive to density information, so when the data clusters have strong edge connectivity, it often fails to cluster such data clusters with distinctly different centers correctly. Therefore, in some datasets, the results obtained by DBSCAN have large differences from the actual situation.

### 4.2. Unsupervised Dataset Experiments

To demonstrate that the adaptive ability of the method in this paper is still very competitive on unsupervised datasets, we test the BP algorithm and our SANBP algorithm using artificial data [3]. On this spherical dataset with a large number of outlier points, SANBP algorithm achieves more intuitive and significant improvement in clustering results. In addition to the clustering results, SANBP algorithm is able to perform neighborhood analysis adaptively according to different data distribution characteristics, thus making the initial boundary points of border peeling superior to the BP clustering algorithm in terms of number and location.

**Figure 4:**
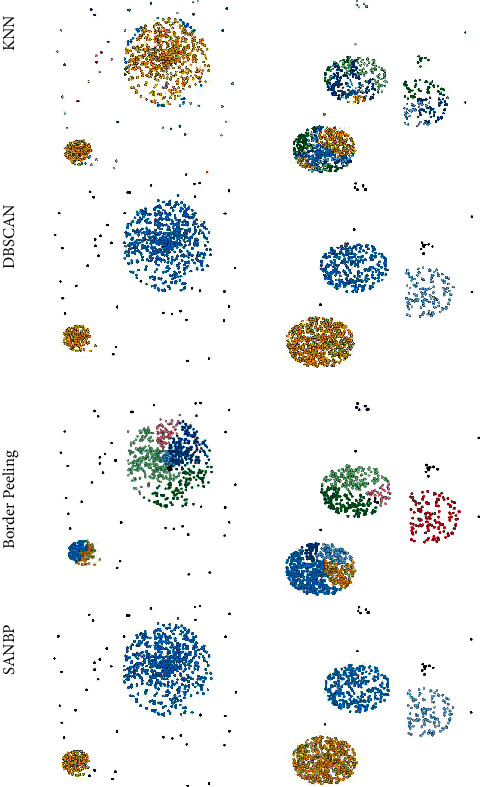
Comparison with BP clustering algorithm on artificial_data datasets.

The experimental results on the datasets data_DBSCAN and artificial_data are shown in Figure 4. In the datasets, SANBP algorithm which is proposed in this paper reflects strong adaptive performance, correctly recovers the original number of clusters, and has good results in the determination of outlier points. As a comparison, the BP algorithm did not obtain effective clustering results, and the final outlier points were poorly delineated. This result also shows that SANBP algorithm has the ability to be adaptive on different shapes and different numbers of datasets, and this adaptive method of generating neighborhood parameters can better determine the initial boundary points in the process of border peeling, as well as optimize the final clustering results and the delineation of outlier points to a great extent.

Experiments show that in unsupervised datasets with a large number of outlier points, SANBP algorithm clusters much better than the BP algorithm in several areas such as the number of clusters and the quality of clusters.

### 4.3. Experiments on Large Datasets

To further validate the performance of our technique on large datasets, we generated large sets by extracting feature vectors generated by convolutional neural networks (CNN) that were trained separately on MNIST [28]. MNIST is a well-known handwritten digit image dataset which consists of 70000 labeled images of handwritten digits divided into a training set of 60,000 images and a test set of 10,000 images. To verify the adaptive nature of the method in this paper, we randomly generated three datasets (D1, D2, and D3) for radius size of 120, 130, and 140, each including a few thousand or so elements, by randomly generating datasets with unknown number of clusters and variable shapes, and then downscaled the sampled data to 30 by randomly sampling the data in different radii on the large dataset. In this paper, we run the dataset for each radius value 10 times and take the average value.

**Figure 5:**
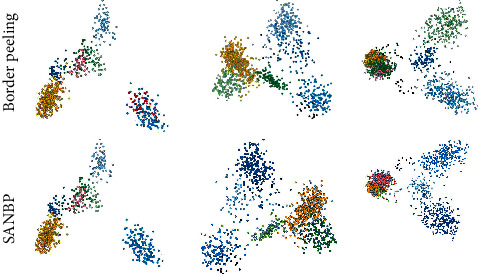
Embedding results of selected samples of MNIST features generated by CNN in 2 d.

As shown in Figure 5, the three datasets are run on the BP algorithm and SANBP algorithm for ten times with the best results, and then the selected samples are embedded into a 2-dimensional coordinate system. From the figure, we can see that the three datasets have different shapes, and the method in this paper can still use the idea of natural neighbor to generate logarithmic natural eigenvalues adaptively, which makes the initial boundary point selection of border peeling more universal, and the method in this paper is more reasonable in the determination of outlier points. In particular, the clustering effect in D2 and D3 datasets shows better competitiveness than the original BP clustering algorithm.

From the experimental results in Table 2, we can see that the results produced by SANBP algorithm have a good representation on the high-dimensional dataset. Although SANBP algorithm is slightly lower than the BP clustering algorithm on dataset D1, the difference in the final clustering evaluation index is not large. On datasets D2 and D3, the performance of each method in this paper exceeds that of the BP clustering algorithm, and the final clustering results are better.

### 4.4. Algorithm Performance and Operational Details

The SANBP algorithm implemented in this paper is written using the Python language, and the time complexity of the BP clustering algorithm is $O\left(T\cdot \left(k\cdot n+{\tilde{f}}_{\text{knn}}\right)\right)$, where ${\tilde{f}}_{\text{knn}}$ is the asymptotic complexity of the KNN method for the dataset; the time complexity of SANBP algorithm in this paper is slightly higher than the time complexity of the BP clustering algorithm, with a time complexity of $O\left(n\;\log\;n+T\cdot \left(k\cdot n+{\tilde{f}}_{\text{knn}}\right)\right)$. The KD-tree is constructed when natural neighbors are used, making the time complexity in finding log natural neighbors *O*(*n*  log  *n*).

## 5. Conclusions

In this paper, we propose a self-adaptive neighborhood border peeling clustering algorithm (SANBP), which can solve the problem of parameter adaption such as the number of clusters and neighborhood parameters in clustering algorithms. Firstly, SANBP algorithm adapts to datasets of different shapes through a robust natural search algorithm. Secondly, SANBP generates self-adaptive neighborhood with the distribution pattern of the dataset and finally uses self-adaptive neighborhood to replace the fixed neighborhood parameters. When the dataset reaches the logarithmic natural stable state, the self-adaptive neighborhood of each point in the dataset is obtained simultaneously and reflects the distribution of the data. SANBP algorithm can establish more ideal initial boundary points according to the different shapes of the dataset, which makes the association between boundary points and core points more reasonable in each iteration of border peeling and finally results in good clusters.

Unlike other clustering algorithms, the method in our paper uses self-adaptive neighborhood and can generate high-quality initial boundary points according to different shaped datasets. The experiments also show that the initial boundary point distribution has an important impact on the final clustering effect. In the whole experiment, whether on the original experimental dataset of BP clustering algorithm or on a large number of other datasets with different shapes, our algorithm is more competitive than the original BP clustering algorithm.

Although SANBP algorithm has achieved satisfactory results in terms of parameter adaptation and clustering results, it still has room for further improvement. In the follow-up work, we will focus on the improvement of the algorithm to make it better adapted to the performance in semisupervised datasets, and the algorithm will focus more on the application to specific practical problems for generalization. In terms of neighbor relationship, we will explore the idea of optimizing natural neighbors for problems such as stream data overlap and automatic data labelling and try to better apply the natural neighborhood graph of natural neighbors to the clustering algorithm to further improve the search efficiency of the algorithm.

## Figures and Tables

**Algorithm 1:**
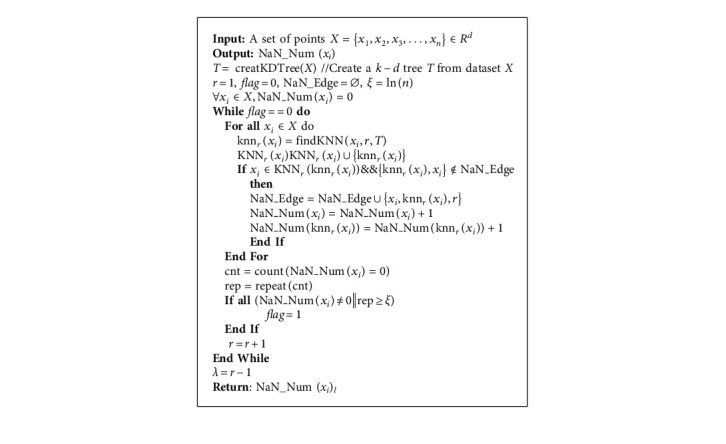
Natural neighbor searching algorithm.

**Algorithm 2:**
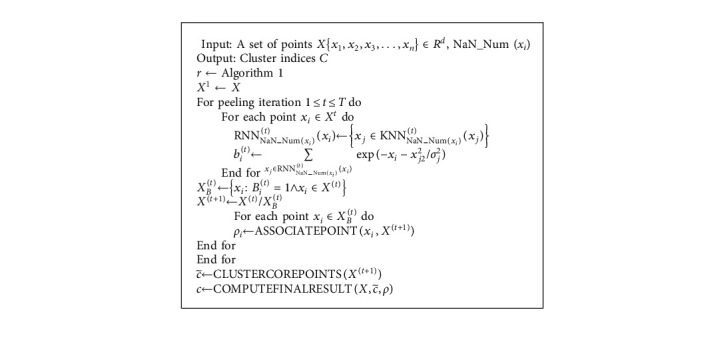
SANBP clustering algorithm.

**Table 1:** Performance comparison (datasets flame, R15, compound, and D31).

	Flame		R15		Compound		D31	
	ARI	AMI	ARI	AMI	ARI	AMI	ARI	AMI
KNN	0.925	0.773	0.909	0.866	0.904	0.702	0.895	0.897
DBSCAN	0.249	0.298	0.912	0.950	0.424	0.410	0.453	0.782
Border peeling	**0.955**	**0.884**	**0.993**	**0.987**	0.646	0.765	0.805	0.912
SANBP	**0.955**	0.857	0.944	0.941	**0.946**	**0.938**	**0.906**	**0.941**

All the bold values identify the best result.

**Table 2:** Comparison with BP clustering algorithm on MNIST dataset samples.

	D1 (*r* = 120, classes = 7)				D2 (*r* = 130, classes = 6)				D3 (*r* = 140, classes = 7)			
	ARI	AMI	Det#	K	ARI	AMI	Det#	K	ARI	AMI	Det#	K
BP	0.977	**0.962**	7.3	20	**0.930**	**0.892**	6.3	20	0.860	0.886	7.7	20
SANBP	**0.980**	0.865	**7.0**	31	0.921	0.865	**6.0**	30	**0.920**	**0.888**	**7.1**	34

All the bold values identify the best result.

## Data Availability

The data used to support the findings of this study are available from the corresponding author upon request.
